# Supplementary figures and images for: From conservation to structure, studies of magnetosome associated cation diffusion facilitators (CDF) proteins in Proteobacteria
Source: PLoS One. 2020 Apr 20;15(4):e0231839. doi: 10.1371/journal.pone.0231839 (PMC7170241; doi:10.1371/journal.pone.0231839)

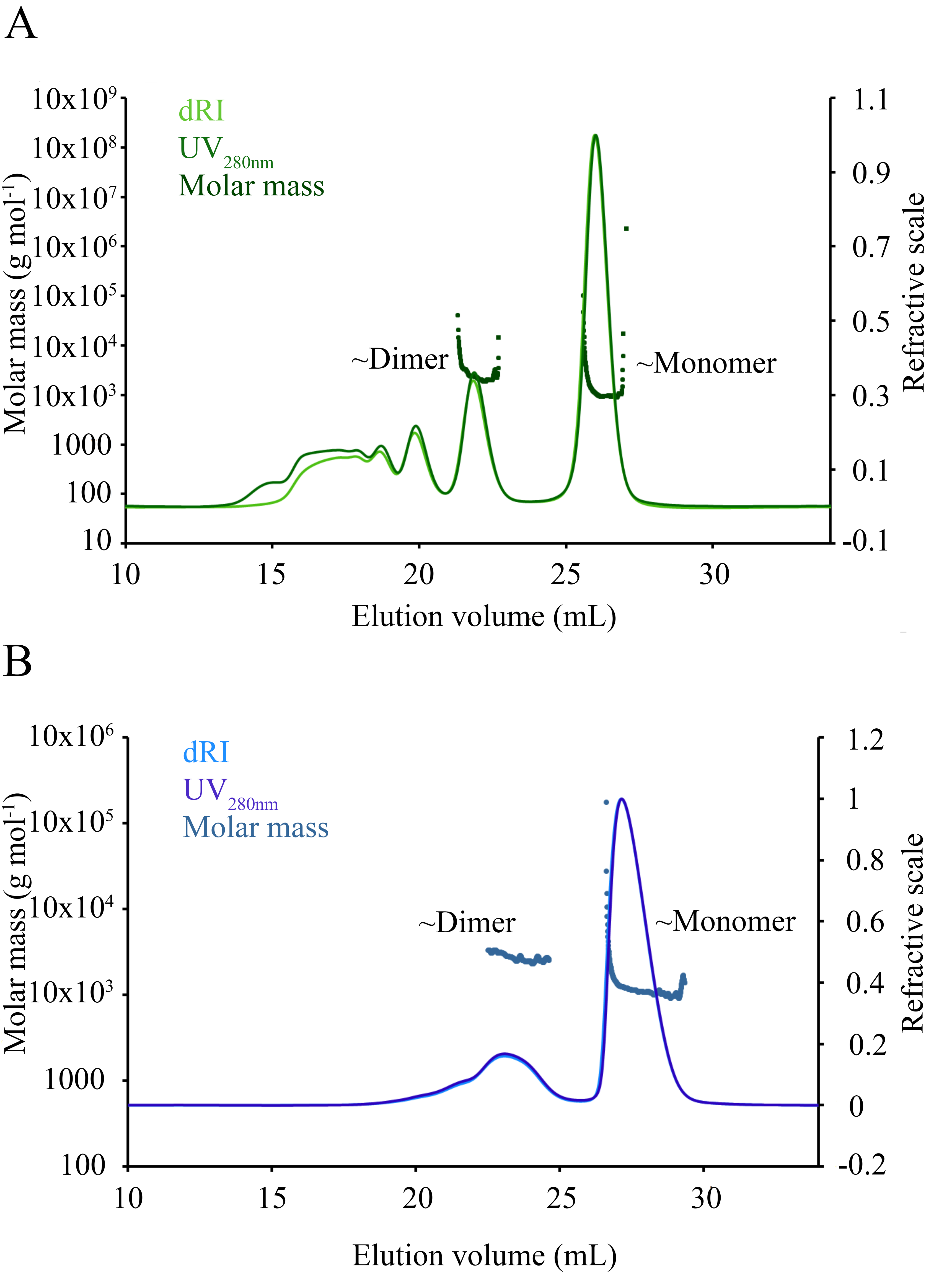

Supplement: S1 Fig — SEC-MALLS chromatogram shows the elution curve of BW-1 CTD proteins, refractive index correlated with UV absorbance at 280nm. Molar mass was calculated. (TIF) [file pone.0231839.s001.tif]

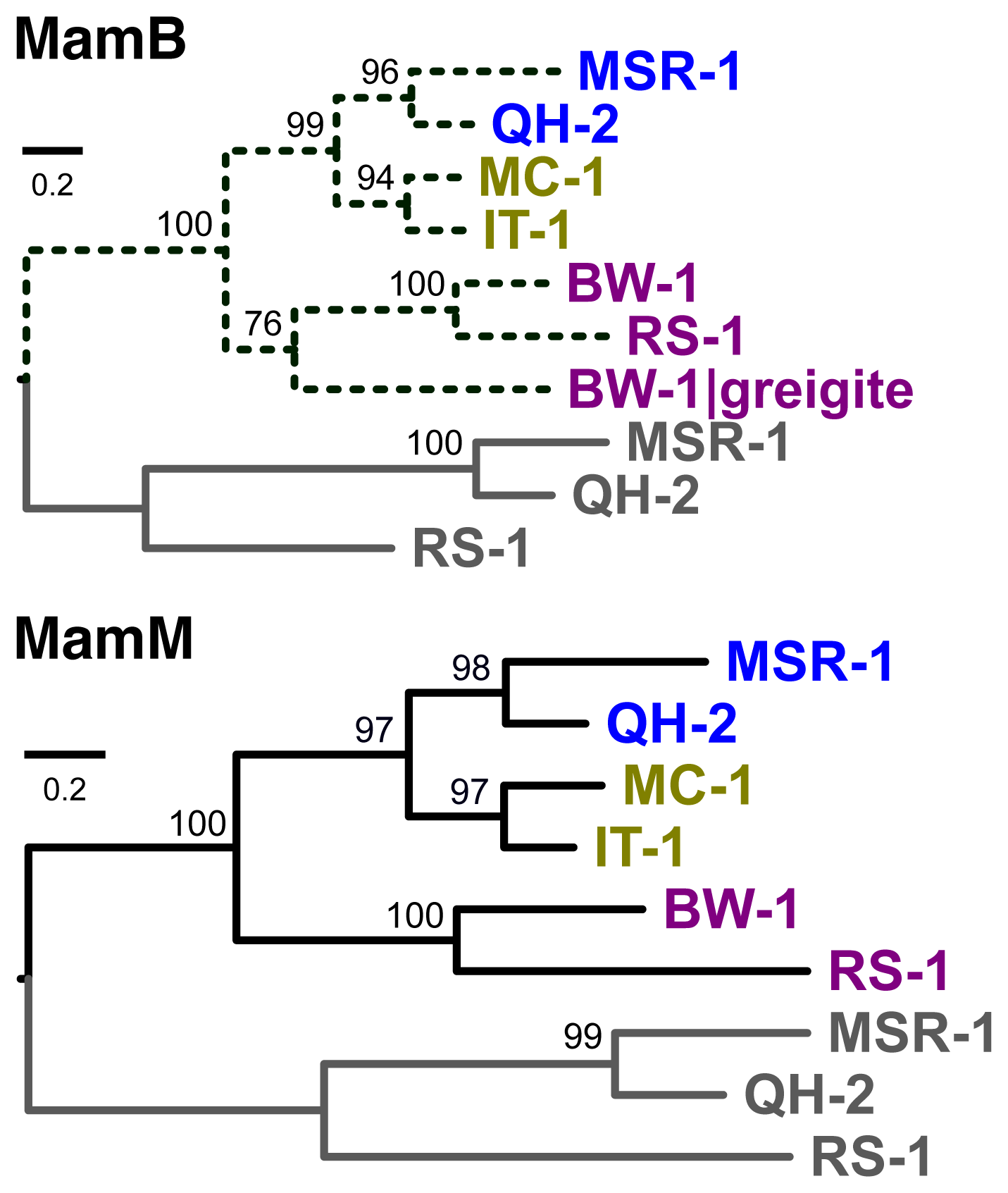

Supplement: S2 Fig — The trees were built using the Maximum-Likelihood method implemented in IQ-TREE and the trimmed alignment of FieF with MamB or MamM sequences detected in the 6 strains. Color of the strain names correspond to their affiliation given in the species tree (Fig 5): Alphaproteobacteria (blue), Ca. Etaproteobacteria (brown) and Deltaproteobacteria (Purple). The branch length represents the number of substitutions per site. The robustness of the tree topology was tested with 500 replicates of a non-parametric bootstrap approach. (TIF) [file pone.0231839.s002.tif]
