# Supplementary material for: From conservation to structure, studies of magnetosome associated cation diffusion facilitators (CDF) proteins in Proteobacteria
Source: PLoS One. 2020 Apr 20;15(4):e0231839. doi: 10.1371/journal.pone.0231839 (PMC7170241; doi:10.1371/journal.pone.0231839)
Supplement: S1 Table — (DOCX) [file pone.0231839.s003.docx]

**S1 Table. small angel x-ray scattering data analysis.**

| **(*a*) Sample details** | | | | |
| --- | --- | --- | --- | --- |
| Organism | *Escherichia coli* | | | |
| Source | *Desulfamplus* *magnetovallimortis* *BW-1* | | | |
| Sequence | MamB BW-1  MamM BW-1 | | | |
| Extinction coefficient |  | | | |
| **(*b*) SAXS data collection parameters** | | | | |
| Source, instrument, and description or reference | BM29-bioSAXS beamline at the European Synchrotron Radiation Facility (ESRF) in Grenoble, France | | | |
| Wavelength | 12.5 keV corresponding to a wavelength of 0.998 Å^-1^ | | | |
| Detector & *q*-measurement range (Å^-1^) | The scattering intensity was recorded using a Pilatus 1M detector, in the interval 0.004 < *q* < 0. 5 Å^-1^ (*q* = 4π sinθ/λ, where 2θ is the scattering angle) | | | |
| Volume, exposure time, number of frames and temperature | 50 μl of the sample was exposed for 2 s, Ten frames were recorded for each sample at 25 °C | | | |
| **(*c*) Software employed for SAXS data reduction, analysis and interpretation** | | | | |
| SAXS data reduction | PRIMUS [1] | | | |
| Calculation of extinction coefficient from sequence | Portparm tool | | | |
| Basic analyses: Guinier, *P*(*r*), Porod volume, volume of correlation | ATSAS, GNOM [2,3] | | | |
| Shape/bead modeling | DAMMIN, DAMMIF [4] | | | |
| Atomic structure modeling | CRYSOL [5] | | | |
| **(*d*) Structural parameters** | | | | |
| Guinier Analysis | | | | |
| Sample | **MamB 1 mg/ml** | **MamB 5 mg/ml** | **MamM 1 mg/ml** | **MamM 5 mg/ml** |
| *I(0)* (nm^-1^) | 7.05±0.031 | 9.49±0.013 | 10.19±0.031 | 15.86±0.0025 |
| *R_g_* (nm) | 1.81±0.14 | 2.0±0.07 | 1.87±0.05 | 2.31±0.25 |
| *qR_g_* range | 0.37-1.30 | 0.41-1.27 | 0.33-1.30 | 0.64-1.30 |
| Quality-of-fit parameter (Auto*R_g_*) | 86 | 95 | 91 | 87 |
| *P*(*r*) analysis | | | | |
| Sample | **MamB 1 mg/ml** | **MamB 5 mg/ml** | **MamM 1 mg/ml** | **MamM 5 mg/ml** |
| *I(0)* | 1.66 | 1.98 | 1.84 | 2.31 |
| *R_g_* (nm) | 1.66 | 1.98 | 1.85 | 2.32 |
| *d_max_* (nm) | 4.27 | 6.0 | 5.52 | 7.10 |
| *q* range (nm) | 0.204-4.41 | 0.204-3.998 | 0.176-4.28 | 0.275-3.46 |
| Quality-of-fit parameter (total estimate from GNOM) | 62.55 | 64 | 66.4 | 65 |
| **(*e*) Shape modeling results** | | | | |
| Sample | **MamB 1 mg/ml** | **MamB 5 mg/ml** | **MamM 1 mg/ml** | **MamM 5 mg/ml** |
| DAMMIF (default parameters, 20 calculations) | | | | |
| q range for fitting (nm^-1^) | 0.0204-4.415 | 0.0204-0.3998 | 0.0176-0.428 | 0.0275-0.346 |
| Symmetry | P_1_ | P_1_ | P_1_ | P_1_ |
| χ^2^ range | 6.192 | 11.180 | 22.89 | 27.32 |
| NSD (standard deviation), No. of clusters | 0.651±0.088, 10 | 0.656±0.05, 10 | 0.739±0.05, 10 | 0.850±0.052, 10 |
| Resolution (from SASRES) (Å) | 27±2 | 27±2 | 21±2 | 28±2 |
| M estimate as 0.5 volume of models (Da) | 11105 | 14939 | 12438 | 20808 |
| DAMMIN (with default parameters) | | | | |
| *q* range for fitting (nm) | 0.0204-4.415 | 0.0204-0.3998 | 0.0176-0.428 | 0.0275-0.346 |
| Symmetry | P_1_ | P_1_ | P_1_ | P_1_ |
| χ^2^ | 0.742 | 1.029 | 0.685 | 1.003 |
| **(*f*) Atomistic modeling** | | | | |
| Sample | **MamB 1 mg/ml** | **MamB 5 mg/ml** | **MamM 1 mg/ml** | **MamM 5 mg/ml** |
| CRYSOL (with default parameters) | | | | |
| Crystal structure | 6QFJ | | 6QEK | |
| Predicted *R_g_* (Å) | 15.88 | 16.51 | 14.92 | 19.60 |
| Vol (Å), Ra (Å), Dro (e Å^-3^) | 12959, 1.800, 0.05 | 12959, 1.400, 0.075 | 11640, 1760, 0.075 | 23095, 1.800, 0.075 |
| χ^2^ | 1.545 | 29.989 | 4.618 | 63.782 |

1. Konarev P V, Volkov V V, Sokolova A V, Koch HJ, Svergun DI. PRIMUS : a Windows PC-based system for small- angle scattering data analysis. 2003;1277–82.

2. Franke D, Petoukhov M V, Konarev P V, Panjkovich A. computer programs ATSAS 2. 8: a comprehensive data analysis suite for small-angle scattering from macromolecular solutions. 2017;1–14.

3. Svergun DI. Determination of the regularization parameter in indirect-transform methods using perceptual criteria. J Appl Crystallogr. 1992;25(pt 4):495–503.

4. Franke D, Svergun DI. DAMMIF, a program for rapid ab-initio shape determination in small-angle scattering. 2009;342–6.

5. Barberato C, Koch MHJ, Molecular E, Outstation H. CRYSOL - a Program to Evaluate X-ray Solution Scattering of Biological Macromolecules from Atomic Coordinates. 1995;768–73.
