# Supplementary material for: From conservation to structure, studies of magnetosome associated cation diffusion facilitators (CDF) proteins in Proteobacteria
Source: PLoS One. 2020 Apr 20;15(4):e0231839. doi: 10.1371/journal.pone.0231839 (PMC7170241; doi:10.1371/journal.pone.0231839)
Supplement: S2 Table — (DOCX) [file pone.0231839.s004.docx]

**S2 Table. Crystallization of BW-1 MamB and MamM CTDs.**

| PDB code | 6QFJ | 6QEK |
| --- | --- | --- |
| Protein | MamB CTD BW-1 +His | MamM CTD BW-1 methylated |
| **Data collection** | BM-14 - ESRF | ID23-1 - ESRF |
| Space group | P1 | P6_1_ |
| Condition | 20% PEG 3,350 0.1M Tris pH 8.6  0.2M Ammonium acetate | 25% PEG 3,350 0.1M BIS-TRIS  pH 5.5  0.2M Sodium chloride |
| Cryo protectant | 50% PEG 3,350 | 1µl from condition |
| Protein concentration (mg/ml) | 20 | 16 |
| Crystallization type | Vapor diffusion sitting drop | Vapor diffusion sitting drop |
| Ramachandran statistics**^Φ^** | 99.79% | 96.67% |
|  | 0.21% | 2% |
|  |  | 1.33% |
| Missing residues | B: Ser187  C: Ser187  D-F: Ser187 His188 Met189 | A: Arg301, Asp302, Arg314, Lys315  B: Gly240, Glu251, Ile264, Gly 265, Gln266, Lys267, Arg314, Lys315 |

**(**Φ) A- Fully-allowed region and PEG-Polyethylene glycol.
