# Supplementary material for: From conservation to structure, studies of magnetosome associated cation diffusion facilitators (CDF) proteins in Proteobacteria
Source: PLoS One. 2020 Apr 20;15(4):e0231839. doi: 10.1371/journal.pone.0231839 (PMC7170241; doi:10.1371/journal.pone.0231839)
Supplement: S3 Table — (DOCX) [file pone.0231839.s005.docx]

**S3 Table. Data collection and refinement statistics.**

| PDB code | 6QfJ | 6QEK |
| --- | --- | --- |
| Protein | MamB BW-1 CTD | MamB CTD |
| **Data collection** | BM-14 - ESRF | ID-23-1 - ESRF |
| Space group | P1 | P6_1_ |
| Cell dimensions |  |  |
| *a*, *b*, *c* (Å) | 46.97, 47.07, 74.19 | 46.93, 46.93 47.01, 130.22 |
|  α, β, γ (°) | 74.2, 84.8, 84.2 | 90, 90, 120 |
| Resolution (Å) | 2.13 (2.19) | 1.951(1.85) |
| *R*sym or *R*merge | 0.068 (0.496) | 0.057 (1.944) |
| *I* / σ*I* | 23.7 (2.25) | 11.4 (0.6) |
| *CC 1/2* |  | 99.8 (13.0) |
| Completeness (%) | 98.4 (97.0) | 99.7 (99.7) |
| Redundancy | 3.9 (3.8) | 4.9 (4.8) |
| Wavelength (Å)  Total No. of observation | 0.932 | 0.977  67310 |
| No. unique reflections | 3384 | 13784 |
| **Refinement** |  |  |
| Resolution (Å) | 2.13 | 1.95 |
| *R*work / *R*free | 0.201/0.234 | 0. 206/ 0.271 |
| No. atoms |  |  |
| Protein | 4223 | 1203 |
| Ligand/ion |  |  |
| Water | 155 | 87 |
| *B*-factors |  | 43.580 |
| R.m.s. deviations |  |  |
| Bond lengths (Å) | 0.017 | 0.019 |
| Bond angles (°) | 1.838 | 2.016 |

Values in parentheses are for the highest resolution shell. One crystal was used per data set. Data were collected at 100 K for all crystals. Data collections were performed at beamlines BM-14 and ID23-1 at the European Synchrotron Radiation Facility (ESRF), Grenoble, France and at beamline P-13 at the Deutsches Elektronen-Synchrotron (DESY), Hamburg, Germany.
