# Supplementary material for: From conservation to structure, studies of magnetosome associated cation diffusion facilitators (CDF) proteins in Proteobacteria
Source: PLoS One. 2020 Apr 20;15(4):e0231839. doi: 10.1371/journal.pone.0231839 (PMC7170241; doi:10.1371/journal.pone.0231839)
Supplement: S4 Table — (DOCX) [file pone.0231839.s006.docx]

**S4 Table. Bacterial strains, plasmids, and genomes**.

| ***Strains /Plasmids/*** ***Genomes*** | ***Important features/Sequences/Ref ID*** | ***Source or reference*** |
| --- | --- | --- |
| ***E. coli*** |  |  |
| Rosetta TM (DE3) | F^-^ *ompT hsdS*_B_(r_B_^-^ m_B_^-^) *gal dcm* (DE3) pLysSRARE (Cam^R^) | Novagen® |
| ***Plasmids*** |  |  |
| pET28a-*mamB* BW1-CTD | Kn^R^, N-terminal His•Tag®/thrombin with *mamB* BW1-CTD | this study/ Biomatik© |
| pET28a-*mamM* BW1-CTD | Kn^R^, N-terminal His•Tag®/thrombin with *mamM* BW1-CTD | this study/ Biomatik© |
| ***Genomes*** |  |  |
| ***Acidithiobacillia*** |  |  |
| *Acidithiobacillus ferrooxidans* ATCC23270 | GCA_000021485.1 |  |
| *Acidithiobacillus caldus* ATCC51756 | GCA_000175575.2 |  |
| ***Alphaproteobacteria*** |  |  |
| *Caulobacter vibrioides* | GCA_000006905.1 |  |
| *Mesorhizobium loti* MAFF303099 | GCA_000009625.1 |  |
| *Brucella suis* 1330 | GCA_000223195.1 |  |
| *Parvibaculum lavamentivorans* DS-1 | GCA_000017565.1 |  |
| *Methylobacterium extorquens* PA1 | GCA_000018845.1 |  |
| *Hyphomonas neptunium* ATCC15444 | GCA_000013025.1 |  |
| *Rhodobacter sphaeroides* ATCC17025 | GCA_000016405.1 |  |
| *Rhodospirillum rubrum* ATCC11170 | GCA_000013085.1 |  |
| *Magnetospirillum gryphiswaldense* MSR-1 | GCA_000513295.1 |  |
| *Magnetospirillum magneticum* AMB-1 | GCA_000009985.1 |  |
| *Magnetospira sp.* QH-2 | GCA_000968135.1 |  |
| *Gluconobacter oxydans* 621H | GCA_000011685.1 |  |
| *Rhodospirillum centenum* SW | GCA_000016185.1 |  |
| *Sphingomonas wittichii* RW1 | GCA_000016765.1 |  |
