# Supplementary material for: From conservation to structure, studies of magnetosome associated cation diffusion facilitators (CDF) proteins in Proteobacteria
Source: PLoS One. 2020 Apr 20;15(4):e0231839. doi: 10.1371/journal.pone.0231839 (PMC7170241; doi:10.1371/journal.pone.0231839)
Supplement: Attachment — Submitted filename: Response to Reviewers .docx [file pone.0231839.s008.docx]

Dear Prof. Eric

Thank you for your evaluation and the positive comments. Sorry for the late revision but we were delayed due to the Corona situation. Please find a corrected article with modified analyses based on the reviewer comments. During this analysis we gained a more profound help from Prof Guy Perriere and added him as an additional author to this paper.

Please find below point by point answers to the reviewer comments.

Yours Raz

We would like to thank all the reviewers for helping us in improving the article and we appreciate the time you spent on this evaluation.

Reviewer #1: This manuscript by Keren-Khadmy et al for the first time characterizes the cation diffusion facilitator MamB and MamM proteins and CTD crystal structures from a proteobacterial magnetotactic bacterium. While the overall structure of these proteins is similar to those of previous CDF-CTD structures their oligomeric states differ. In addition to regular dimeric forms also monomers were observed. Interestingly, also metal binding site aa compositions differ from previous reports.
The experiments are well performed and presented and deserve publication in Plos ONE. However, a few shortcomings (listed below) should be addressed before publication.

L59: „several chains“ – not true for all MTB

Agree. was change to *“Chains of aligned magnetosomes…”*

L61: oxic-anoxic transition zone better than microoxic transition zone

was change accordingly.

LL61: “Most of the genes crucial for the magnetosome biogenesis are specific to MTB” - might be misleading since homologs to mam genes exist outside of MTB as well.

Was removed from the text. *“Most of the genes crucial for the magnetosome biogenesis are clustered in a conserved region within the bacteria genome”*

L63: the bacterial genome of their genomes (?)

was change accordingly.

LL65-67: MamB and MamM are conserved among all MTB (including more phyla than listed here).

Correct, but on the one hand, we are talking about MTBs in which magnetosomes are composed mostly from magnetite. And on the other hand, in this paper we focused at the proteobacteria therefore we choose not to mention all the groups.

LL67: These sentences imply that BW-1 has MamM and B for greigite formation only. I would recommend to modify the whole paragraph to clarify the phylogenetic occurrence of MamB and MamM and the presence of two different magnetosome gene clusters in BW-1 (of which each contains mamB/mamM homologs).

The text was change accordingly.

L72: use bacterial instead of bacterium. Change have been made.
L75: insert a before “cytosolic”. Change have been made.
L79: grammar. Corrected.
LL96: what are the consequences of this alternative dimer packing? Are proteins still functional? The text was edited. *“substitution-mutations within these residues leads to alternated dimeric packing that impaired protein function”.*

LL101: avoid “additionally” and “additional“ within the same sentence. Change have been made.
L104: delete class. Change have been made.

LL145: “Third, we assume that in-vivo MamB and MamM local-concentration might be higher as the magnetosome contains only a few protein complexes that involve MamB and MamM.” – How can the MamB and MamM concentration increase at the magnetosome when it contains only a few complexes?

*Third, we assume that in-vivo MamB and MamM local-concentration might be higher as the primary magnetosome contains only a few protein complexes and has a small surface area which make a larger ratio of protein to surface relative to other CDF in the general cytoplasmic membrane, that mostly involve MamB and MamM.* Text was clarified.

LL151: Previously it was reported that MamM and MamB directly interact with each other. It would therefore also be possible that heterodimeric MamM/B complex are more preferred. This should be at least discussed here.

Change have been made.

*Moreover, MSR-1 alphaproteobacteria MamB-MamM CTDs have previously shown to interact in-vitro and were suggested to assemble as heterodimers [30]. It was further proposed that the magnetosome lumen formed only after reaching critical size and composition of the multi-lipid-protein assembly that also involved MamQ and MamL [33]. In accordance with this research and together with our SAXS results, we expect that in native conditions, MamBBW-1 and MamMBW-1 preferred oligomerization state is a hetro or homodimer.*

LL193: Why does the apo MamM-BW-1 dimer resemble other metal-bound CDF-CTDs?

*As we previously published, it seems that the main fold of the CTD is conserved while the different metals are a local amino acid changes. This leads to a very similar dimerization and structure as it needs to save the main activation mechanism between al CTD containing CDFs.*

LL237: additional trees based on MamM and/or MamB protein sequences would be more informative. Especially since the authors stated in their introduction that their results also indicate that these proteins were under high selection pressure over MTB evolution and likely coevolved in Proteobacteria.

We agree with the reviewer that a protein tree would have been more relevant here. It was planned to address MamB-MamM evolution in another manuscript studying the evolution of all Mam proteins because the study of MamB-MamM revealed quite exciting results that would have deserved its own manuscript. However, following reviewer’s recommendations, we decided to give some insights here, where these results seem to be appropriate. We added several sections in the material and methods sections, the results and perform new analyses in the new figures S2 and 6:

In the introduction, we changed the last conclusion sentence by:

*“Our results also indicate that* MamB *could have emerged from a duplication of* MamM*, which means that the* MamB *ancestor was likely an iron transporter and that its role in magnetosome vesicle invagination and magnetite nucleation emerged secondarily in a magnetotactic ancestral Deltaproteobacteria.”*

In the results section, we modified and added results on MamM-MamB phylogeny:

*“****Structural conservation of CDF proteins in magnetotactic Proteobacteria***

*In agreement with previous phylogenies of Proteobacteria, our phylogenetic tree reconstructed from riboproteins confirmed that the magnetotactic strain BW-1, belongs to a class evolutionary divergent and ancestral to that of magnetotactic strains QH-2 and MSR-1 [36] (Fig 5). Using representative MTB strains of the Alphaproteobacteria, Deltaproteobacteria and* *Candidatus Etaproteobacteria, we inferred the MamB and MamM phylogenies individually using FieF sequences as an external outgroup and observed an apparent congruence between species and trees phylogenies for each protein (Fig. S2). This congruence supports that each paralog has emerged from a common MTB ancestor after a very ancient duplication event. This scenario is thus in agreement with previous studies (ref). However, because the internal branches of the trees were very long, we suspected that this scenario and its good statistical support could have been artifactual and have resulted from the long branch attraction of the Deltaproteobacteria towards the external group. We thus build a tree based on the alignment of the three paralogs to possibly reduce this artefact (Fig. 6). This analysis led to surprising conclusions; both Maximum-likelihood and Bayesian trees support that MamB emerged from a duplication of MamM in Deltaproteobacteria and was then transferred to other Proteobacteria classes by one or several horizontal gene transfers. According to our analyses, emergence of MamB and its secondary function in vesicle invagination were thus more recent than its paralog MamM. Further analyses with a broader taxonomic sampling will be needed to validate this result.*

*Despite the high evolutionary divergence of these homologous proteins, we compared the protein structures obtained for BW-1...”*

The material section corresponding to these new analyses was added:

*“We also investigated the evolutionary relationships between* MamM *and* MamB *of the representative magnetotactic Proteobacteria characterized in this study.* MamB *and* MamM *homologs in MTB strains MSR-1, QH-2, IT-1, MC-1, BW-1 and RS-1 (accessions numbers given in Table SX) were aligned individually and together with the MAFFT software [41], then trimmed using the BMGE 1.12 software [47] (with -b 3 -g 0.5 options). Three maximum-likelihood trees were built with the IQ-TREE software [45] and a substitution model selected using ModelfFinder [46] and the BIC criterion. Since the gene fieF was the genetically closest paralogous gene of the cation diffusion facilitator protein family, three sequences detected in the MTB genomes were used as an external group. For the* MamB*-*MamM *tree showing the evolutionary relationships between the two paralogs, a Bayesian approach was also used to build the phylogeny. For that purpose, we used MrBayes version 3.2.7 [48]. This program explores a large set of substitution models through MCMC sampling (e.g. Poisson, BLOSUM62, WAG). In this approach, each model contributes in proportion to the posterior probability distribution of trees. Site heterogeneity was also taken into account by introducing a Gamma correction with four classes. One million iterations were performed and convergence was estimated using the potential scale reduction factor test. Posterior probabilities at nodes were determined after discarding a burn-in fraction of samples from the chain of 25%.”*

LL261: what intrigued me the most is the apparent “flexibility” of the aa composition of the metal binding sites within the different CDF-CTDs. I think it would be a great benefit for the manuscript if the authors could add paragraph to discuss and analyze metal-binding site variations in a broader way (e.g. is there conservation among certain groups?).

This is a great suggestion that follows our previously published analysis of the metal transport site. However, such an analysis and the amount of data that results from it is beyond the scope of this paper and is deserve a full paper of its own. I do not think we will be able to give a comprehensive analysis or idea in a single paragraph.

Fig.1: Numbering of alignment in A and highlighted metal binding site in B do not match (Position 224 of MamB BW-1 in A is a Val not a Glu as depicted in B).

The numbering of the alignment is according to MamM_MSR-1_ sequence (same for the secondary structure above), therefore there are differences at the numbering within these panels.

Why are there two residues shown on the right monomer in the inset of fig 1B but only one aa label? Crystallographic alternate conformation of E224 at the right monomer.

Bibliography: Ref 19 + 25 and 12 + 30 refer to the same articles

Change have been made.

Reviewer #2: Proteins of the CDF (cation diffusion faciliator) protein family, which are in fact proton-cation-antiporters, transport transition metal cations across biological membranes. The first CDF protein sequences came from yeast (Kamizomo) and a beta-proteobacterium (Nies), the protein family was defined by Saier and the first structure came from E. coli YiiP (Fu). Genes from CDF proteins were found in a gene region responsible for the formation of magnetosomes (Schueler) at a time when first evidence was presented that YiiP may be an iron efflux system re-named as FieF (Grass). Uebe et al described the role of two magnetosome-associated CDFs in magnetite biomineralization.

The authors produced and crystallized the carboxy-terminal domains of the two magnetosome-associated CDFs MamM and MamB from a deltaproteobacterium. These carboxy-terminal domains are compared with each other and the respective domains from two orthologs from alphaproteobacterial. Possible amino acyl residues involved in metal binding and dimerization are discussed. This allows the first direct comparison of a MamM with a MamB from the same organism.

1. Line 47. Many abbreviations are used only a few times and should not be used. Abbreviations of bacterial species names should not be listed as abbreviation since this is standard operation procedure. If listed, they should be in italics. If LB is defined, Luria with capital “L”.

Changes have been made.

2. Line 64. Cite the source for the name CDF or TC category.

Change have been made.

3. Line 108. When mentioned a second time, the genus name of a bacterial species name must be abbreviated.

Change have been made.

4. Line 123. Only genes are expressed, proteins are produced.

Change have been made.

5. Line 127, too many “we” for a scientific paper.

Changes have been made.

*To better characterize protein populations,* *size-exclusion chromatography with* *multi-angle light scattering (SEC-MALS) was used. According to the SEC-MALS, the correlated protein* *molecular weight (MW) was calculated, 9.6 and 21 kDa for MamB_BW-1_ and 11 and 25 kDa for MamM_BW-1_ (S1b Fig).*

6. Line 134, to demonstrate that I have read it: two commas.

Ok

7. Line 139. The hypothesis that the transmembrane part of the CDF contributes to the formation of the dimers comes at line 145. Nevertheless, differentiate here between the references citing dimeric full-length proteins and dimeric isolated domains.

Changes have been made.

8. Line 158 and methods. I know some protein-structure people who have foam around their mouth if a structure was obtained from a protein isolated using a His-tag, because this tag may influence the structure heavily. I would not go that far. Nevertheless, since the His-tag was followed by a thrombin recognition sequence, why was the His-tag not removed after the protein purification? The risk of an artifact would be decreased.

The reviewer is correct. We indeed purified and even cleaved the tag. However, such purified protein without a tag did not yield diffracting crystals. As such, we wrote what work for getting the correct structure and did not related to the things that failed. Previously for other CDFs-CTDs we showed that some CTDs only crystallized with a tag some without and some with both.

9. It seems to be the PLOS format but having the legend within the text is strange and interrupts the reading flow.

Sorry for that, it is mandatory format.

10. Line 190. What are the forces used for the interaction of hydrophobic amino acyl residues such as Val and Ile with the helix breaker Pro and the polar Ser?

Text was modified accordingly. The contacts are complex composition of various contacts.

11. Figure 5. I do not see why the deltas should be ancestral to the alphas.

Phylogenies need to be rooted with the closest monophyletic group that is external to the clade for which we want to infer the phylogeny. The Epsilonbacteraeota are currently used to root the Proteobacteria phylogeny as proposed by recent studies. As explained in the experimental procedures:

*“ This database was completed with the genome of BW-1 strain and that of 7 species representing the Epsilonbacteraeota [38], a novel phylum proposed as ancestral to Proteobacteria [38,39] composed of the Epsilonproteobacteria and the Desulfurellales.”*

Using this relevant external group here, the phylogeny indicates that the Deltaproteobacteria was the first class to have emerged over Proteobacteria diversification, since the topological distance with the root is shorter.

12. Line 274. This seems to be the conclusion. If the main message of this paper is the direct comparison of the structures of the two different magnetosome-associated CDFs, what did we learn? It all looks the same.

13. Line 362. What is the sense of this methods chapter?

In order to reach high ordered crystal of MamM_BW-1_, we preform non-specific lysine methylation that have reduce protein free energy and improved diffraction resolution.

14. Fig. 1. There is no E224 in MamB BW-1 but a Val. Why is the shown residue duplicated in one protomer? Two solutions of the same amino acyl residue?

The numbering of the alignment is according to MamM_MSR-1_ sequence (same for the secondary structure above), therefore there are differences at the numbering within these panels. Crystallographic alternate conformation of E224 at the right monomer.

15. Figure 2. The message of this figure is: no interesting differences.

16. Figure 3. Again, how are these amino acid residues supposed to interact? Van der Waals? Hydrogen bonds? Not from a Val residue.

Addressed in line 190.

Reviewer #3: In this paper, Keren-Khadmy et al, determined crystal structures of C-terminal domains (CTDs) of MamB and MamM from Desulfamplus magnetovallimortis strain BW-1. Overall, this paper is well written contains interesting results.
The authors discussed about structural conservations of CTDs of cation diffusion facilitator proteins in magnetotactic bacteria. The works on structural conservation in magnetosome-associated proteins is still very limited. I think that this paper has value to publish for accumulating molecular information about magnetosome synthesis.
